# Complete Chloroplast Genome Characteristics and Phylogenetic Analysis of *Brassica juncea* L.

**DOI:** 10.3390/ijms27062882

**Published:** 2026-03-23

**Authors:** Shenyue Tang, Juan Liu, Ziyi Zhu, Xingcai An, Junyuan Dong, Xiahong Luo, Changli Chen, Tingting Liu, Lina Zou, Shaocui Li, Xia An

**Affiliations:** 1 Zhejiang Xiaoshan Institute of Cotton & Bast Fiber Crops, Zhejiang Institute of Landscape Plants and Flowers, Zhejiang Academy of Agricultural Sciences, Hangzhou 311251, China; 13587929160@163.com (S.T.); 13456319193@163.com (Z.Z.); xcan2001str@163.com (X.A.); 13964552682@163.com (J.D.); luoxh@zaas.ac.cn (X.L.); chenchangli@zaas.ac.cn (C.C.); liutt@zaas.ac.cn (T.L.); zoulina1991@yeah.net (L.Z.); lishaocui@zaas.ac.cn (S.L.); 2 College of Environment and Resources, College of Carbon Neutrality, Zhejiang A&F University, Hangzhou 311300, China; liujuan@zafu.edu.cn; 3 School of Agriculture, Yunnan University, Kunming 650500, China

**Keywords:** *Brassica juncea*, chloroplast genome, phylogenetic analysis, feature analysis

## Abstract

Yeyong mustard is a mustard vegetable belonging to the Brassicaceae family and the *Brassica* genus. This study assembled, annotated, and analyzed the chloroplast genome of *Brassica juncea* L., aiming to clarify its systematic evolutionary relationship with other cruciferous plants. The study used the Illumina NovaSeq 6000 platform to sequence the entire chloroplast genome of leaf mustard, and systematically analyzed its genome structure, repeat sequences, nucleic acid diversity, and codon preferences using bioinformatics methods. At the same time, the phylogenetic relationships were constructed by combining the leaf chloroplast genome sequences of other cruciferous plants. The results showed that the chloroplast genome of leaf mustard had a total length of 153,490 bp and a GC content of 36.36%, exhibiting a typical tetrad structure; a total of 132 coding genes were annotated, including 87 mRNA genes, 37 tRNA genes, and eight rRNA genes, and no pseudogenes were found. Codon preference analysis shows that leucine (Leu) has the highest frequency of use, with 32 codons having a relative synonymous codon usage (RSCU) greater than 1, mostly ending in A or U; there are 37 scattered repetitive sequences and 315 simple repetitive sequences in the genome. Ka/Ks analysis showed that the chloroplast genes of leaf mustard were subjected to purification selection as a whole, while genes such as *nadhF* and *petD* showed positive selection, which is speculated to be related to adaptive evolution. The results of the phylogenetic analysis further confirm that the chloroplast genome of leaf mustard has a typical tetrad structure and is relatively conserved. It is most closely related to mustard greens in terms of evolutionary relationship, followed by Brassica plants such as nori and turnip, and is also closely related to Brassica plants such as European rapeseed. This study elucidated the conservative characteristics and evolutionary patterns of the chloroplast genome in mustard leaves, providing theoretical support for the phylogenetic research of the Brassicaceae family and the development and utilization of germplasm resources.

## 1. Introduction

Mustard (*Brassica juncea* L.) is native to China and belongs to the Brassicaceae family and the Brassica genus. It is a biennial herbaceous plant and has become an important vegetable crop, oil crop, and seasoning crop in the world [1]. In 1935, Korean Japanese agronomist Changchun Xuyong proposed that all Brassica crops, including the remaining widely distributed wild species, could be classified into six species. Among these six species, the three with fewer chromosomes—*Brassica nigra* with eight pairs of chromosomes, *Brassica oleracea* with nine pairs of chromosomes, and *Brassica rapa* with 10 pairs of chromosomes—are the “basal species”. *Brassica juncea* (AABB) is a heterozygous tetraploid plant formed naturally by distant hybridization of diploid Chinese cabbage (*Brassica rapa*, AA) and black mustard (*Brassica nigra*, BB) [2]. Mustard, as a vegetable, oil crop, and seasoning crop, is widely cultivated worldwide. The annual cultivation area of mustard in China is about 10,000 km^2^, with an annual output of 45 million tons and an output value of over 200 million yuan. These data illustrate the extensive scale of the mustard industry as well as its crucial role in the agricultural sector and public welfare [3]. Mustard greens are rich in protein, fiber, ascorbic acid, and various vitamins, and have functions such as refreshing the mind, detoxifying and reducing swelling, broadening the intestines and stimulating appetite, and promoting digestion and bowel movements [4]. According to their different edible organs, mustard greens can be divided into root mustard greens, stem mustard greens, leaf mustard greens and mustard greens for bolting. Among them, leaf mustard, such as large leaf mustard, is a high-quality raw material for making pickled Chinese cabbage. In the “Guang Qun Fang Pu · Vegetable Recipe V”, it was written that “there is a dish named potherb mustard in Siming, which is unique because of its deep snow and frozen damage.” Potherb mustard is not only cold 56 resistant, but also can be used to make Meigan cai. Leaf mustard is not only suitable for fresh consumption and processing, but also for cooking, frying, pickling, or sun drying [5]. Therefore, strengthening the basic research and genetic breeding work of leaf mustard, exploring the excellent germplasm resources and genes of leaf mustard, has important theoretical and practical significance for promoting the sustainable development of the mustard vegetable industry.

Plant chloroplasts are semi-autonomous organelles with a bilayer membrane structure [6], which directly determine crop yield by converting light energy into ATP and carbohydrate energy. Drought, floods, salt alkali, extreme temperatures, nutritional imbalances, pathogens, viruses and other adverse environmental factors can significantly reduce plant yield once they interfere with plant photosynthetic function. Chloroplasts have three types of membrane structures: double layered membranes (inner and outer membranes) and thylakoid membranes. Each membrane is equipped with specific ion channels and transporters, which can efficiently mediate the transmembrane transport of nutrients, solutes, and metabolites [7]. In recent years, with the rapid development of high-throughput sequencing technology, significant progress has been made in the study of plant chloroplast genomes. Chloroplast DNA is generally a double-stranded circular molecule, and the chloroplast genomes of most higher plants are highly conserved tetrad structures [8]. It consists of two reverse repeat regions (IRs) and separated large single-copy regions (LSCs) and small single-copy regions (SSCs), with a length typically ranging from 107 to 218 kb. It has the characteristics of a highly conserved genome, slow evolution, and maternal single parent inheritance [9], and plays a significant role in plant systematic evolution research, photosynthetic molecular mechanism analysis, and genetic engineering [10].

Brassicales, as one of the largest orders of angiosperms, exhibits significant diversity in morphology and habits, and holds important ecological and economic significance. The Brassicaceae family is very large, consisting of approximately 351 genera and 3977 species [11]. *Brassica*, as a key member of the Brassicaceae family, includes important vegetable and oil crops such as *B. campestris*, *B. oleracea*, *B. juncea*, and *B. napus* [12]. As an important member of the Brassica genus, mustard has undergone long-term artificial selection and environmental changes, resulting in many varieties. Based on the stable and significant differences in morphological structure, divided mustard into five categories: root mustard, stem mustard, leaf mustard, mustard greens for bolting and seed mustard, with 17 varieties [13]. However, the phylogenetic position of leaf mustard in the Brassicaceae family and its evolutionary relationship with related species are still unclear. The chloroplast genome can provide rich genetic information for plant evolution research. In this study, a representative leaf mustard variety was selected, and high-throughput sequencing technology and bioinformatics methods were used to sequence, assemble, and annotate its chloroplast genome, and to analyze its structural characteristics and functional genes in depth. Meanwhile, through phylogenetic analysis, the evolutionary status of leaf mustard in the Brassicaceae family and even the Brassicales order was clarified. The results of this study will provide an important theoretical basis for systematic development research, germplasm resource protection and utilization of cruciferous plants.

## 2. Results

### 2.1. Basic Characteristics of the Chloroplast Genome of Brassica juncea *L.*

**Figure 1:**
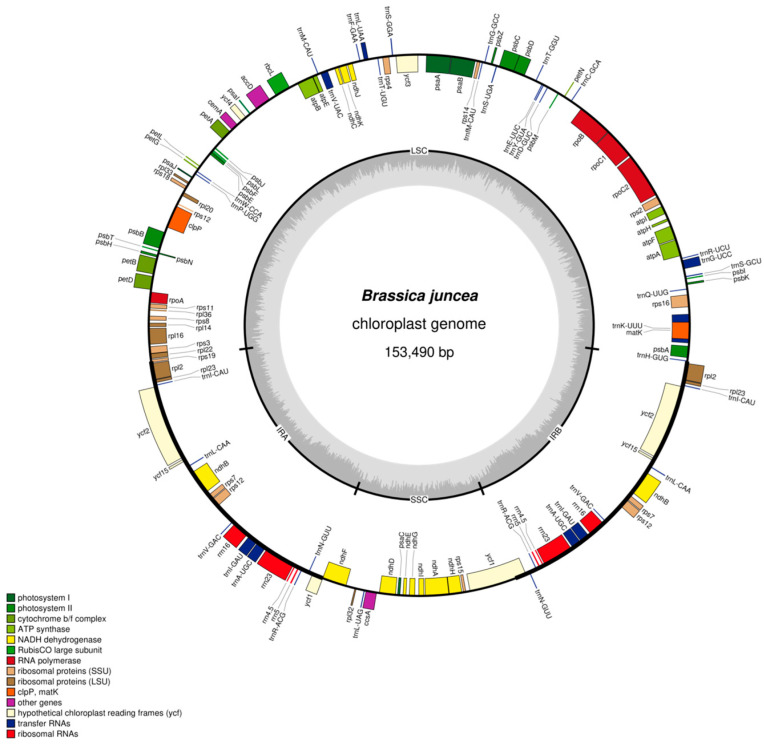
Map of *Brassica juncea* L. chloroplast genome.

In terms of materials and methods, I selected ‘Chicken Crown Snow Cabbage’ leaf mustard as the experimental variety for sequencing and obtained the following results. The chloroplast genome of mustard leaves exhibits a classic tetrad structure, consisting of four parts: large single-copy regions (LSCs) and small single-copy regions (SSCs), inverted repeat sequence a (IRa) and inverted repeat sequence b (IRb), with respective lengths of 83,293 bp, 17,775 bp, 26,211 bp, and 26,211 bp (Figure 1, Table 1). The chloroplast genome length of leaf mustard is 153,490 bp. Among them, A, C, G, T, and GC account for 31.35%, 18.51%, 17.85%, 32.29%, and 36.36% of the total, respectively. The GC content is relatively high among them. The GC content of IRa and IRb is 42.34%, higher than that of the LSCs (34.12%) and SSCs (29.20%) (Table 1).

The genome has the highest proportion of GC in the single-copy region (LSC) at 34.46%, with a quantity of 31,130 bp; in the small single-copy region (SSC), A has the highest proportion at 35.49%, with a quantity of 6309 bp; in the reverse repeat sequence a (IRa), GC has the highest proportion at 42.34%, with a quantity of 11,098 bp; in the reverse repeat sequence b (IRb), GC has the highest proportion at 42.34%, with a quantity of 11,098 bp (Table 1).

### 2.2. Functional Annotation of Chloroplast Genome in Brassica juncea *L.*

In the chloroplast genome of mustard greens, a total of 132 genes were identified, including 87 mRNA genes, 37 tRNA genes, and eight rRNA genes, and no pseudogenes were found. These genes affect the photosynthesis and self-replication within chloroplasts, determining the normal functioning of various life activities in chloroplasts. In addition, five genes involved in other functions (such as mature enzymes, proteases, etc.) and five genes with unknown functions (*ycf* genes) were annotated. (The five genes with unknown functions are *ycf1 (2)*, *ycf15 (2)*, *ycf2 (2)*, ycf3 **, and *ycf4*, located in the last row of Table 2).

A total of 44 genes have been identified to affect the progress of photosynthesis. Among the genes that affect chloroplast photosynthesis, there are five genes that affect photosystem I; there are 15 genes that affect photosystem II; and there are 11 genes that affect NADH dehydrogenase, among which the *ndhB* gene has undergone two copies; however, the fact that the *ndhB* gene has undergone two copies does not affect the change in gene quantity. There are six genes that affect cytochrome complexes, six genes that affect ATP synthase, one gene that affects 1,5-diphosphate carboxylase synthesis, and zero genes that affect photosynthetic pigment reductase.

A total of 59 genes were identified to be involved in chloroplast self-replication, including nine genes affecting large subunit ribosome synthesis, 12 genes affecting small subunit ribosome synthesis, four genes for RNA polymerase, four genes for rRNA synthesis, and 30 genes for tRNA synthesis.

In the chloroplast genome of mustard greens, the copy number of 57 mRNAs and 21 tRNAs is one, and the copy number of 11 mRNAs, 14 tRNAs, and four rRNA is two. The number of introns in 10 mRNAs and eight tRNAs is one, and the number of introns in two mRNAs is two (Table 2).

### 2.3. Analysis of Codon Preference

A systematic analysis was conducted on the codon usage characteristics of the chloroplast genome of mustard greens. The results showed that there were 22,765 codons, of which 22,686 codons were involved in amino acid coding (the termination codon Ter included 79 codons that were not included in the statistical category), forming 21 types of amino acids. The termination codon (Ter) has three nucleotide compositions: UAA has a total of 48 codons with a preference of 1.8228; UAG has a total of 19 codons with a preference of 0.7215; and UGA has a total of 12 codons with a preference of 0.4557.

Among the corresponding codons of various amino acids, the codon encoding leucine (Leu) has the highest frequency of use, with a total of 2416 codons; the number of codons encoding isoleucine (Ile) and serine (Ser) is second, with 1976 and 1694 codons, respectively.

Further analysis of synonymous codon usage (RSCU) shows that there are 28 codons with RSCU > 1, including 26 codons ending in A or U; there are 35 codons with RSCU < 1 and 32 codons ending in G or C. It is worth noting that tryptophan (Trp) is only encoded by one codon, UGG, with an RSCU value of 1. Among all codons, AUG serves as the starting codon and encodes methionine (Met), with the highest RSCU value of 6.9867. The RSCU values of UUA encoding leucine and GCU encoding alanine are relatively high, at 2.163 and 1.8872, respectively. The RSCU value of GUG encoding methionine is the lowest, at only 0.0133 (Table 3).

Methionine has only been used in AUG (512 times), with a codon preference value of 6.9867, significantly higher than other codons, consistent with its uniqueness as a starting codon. Other codons (such as AUA, AUC, etc.) have not been used, reflecting the specificity of the starting codon.

**Figure 2:**
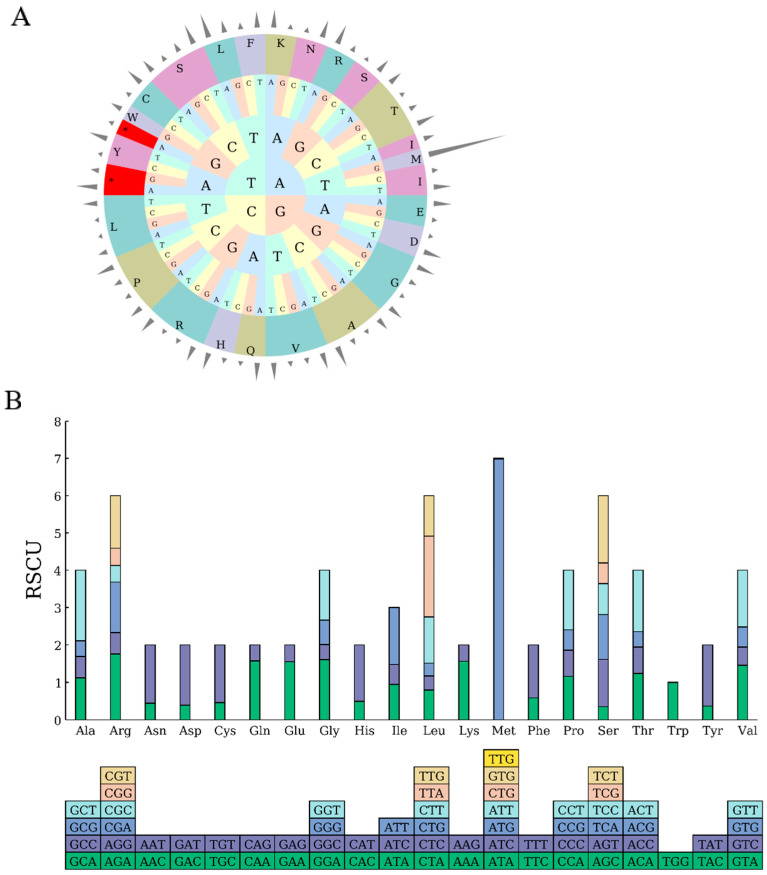
RSCU Pie Chart (**A**) and RSCU Histogram (**B**). Note: The outermost cylinder crepresents the RSCU value, the middle layer consists of amino acids, and the innermost three layers represent codons. Different colors denote different amino acids (abbreviations are labeled on the outer ring, e.g., L for leucine, F for phenylalanine, etc.); the inner letters (A, T, C, G) indicate nucleotides; asterisks (*) mark codons with significant characteristics. Note: The squares below represent all codons encoding each amino acid, while the height of the columns above represents the total sum of RSCU values for all codons.

In addition, by combining the circular graph (Figure 2A) and bar graph (Figure 2B) composed of codons, the distribution of corresponding codons for each amino acid can be visually presented, which plays an important auxiliary role in further analyzing the codon usage patterns of the chloroplast genome of mustard leaves.

### 2.4. Repetitive Sequence Analysis

Simple repeat sequence SSRs are a type of sequence composed of short sequences of one to six nucleotides that are concatenated as repeat units. There are a total of 315 SSRs in the chloroplast genome of mustard greens, including 139 LSCs, 23 SSCs, and 78 IRs. From the composition of genes in different regions, in the LSC region, there are 46 SSRs in the exon, 14 SSRs in the intron, and 79 SSRs in the intergenic region; in the SSC region, there are four SSRs in the exon, zero SSRs in the intron, and 19 SSRs in the intergenic region; and in the IR region, there are 53 SSRs in the exon, one SSR in the intron, and 24 SSRs in the intergenic region. Among these SSRs, single nucleotide repeat types are diverse and abundant, with A repeat numbers ranging from eight to 15 and numbers between one and 43; the number of repetitions for T varies from eight to 22, with quantities ranging from one to 48; and the number of repetitions of C varies from eight to 11, with quantities ranging from one to four. There are also a certain number of dual nucleotide repeats such as AT, TA, etc. There are many types of trinucleotide repeats, such as AAC, AAG, etc. Tetranucleotide repeats also exist in small quantities.

**Figure 3:**
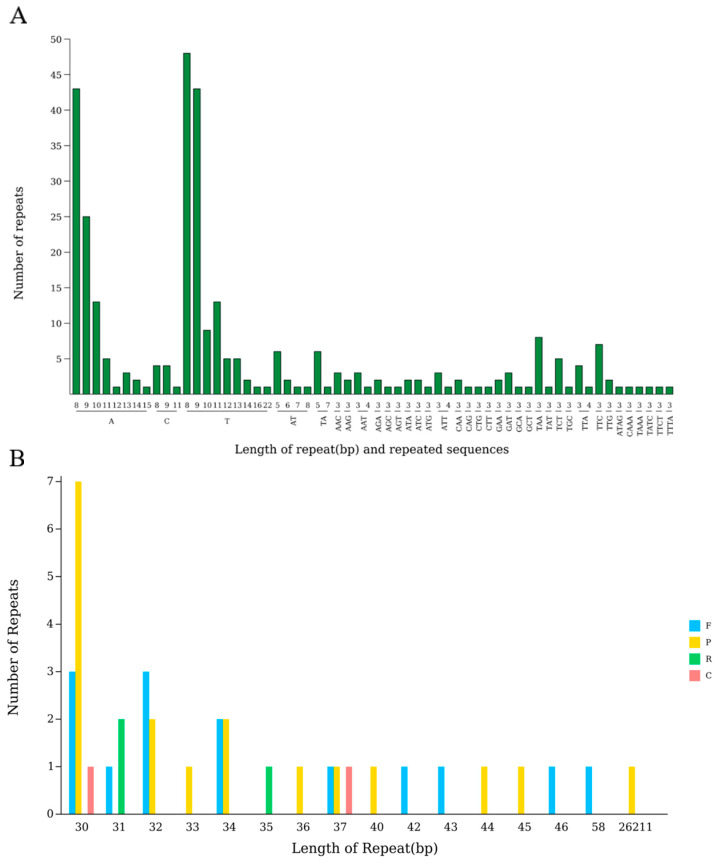
Analysis of simple sequence repeats (**A**) and scattered sequence repeats (**B**) in the chloroplast genome of *Brassica juncea* L. Note: The horizontal axis represents the length of scattered repetitive sequences, while the vertical axis represents the number of scattered repetitive sequences. F denotes forward repeats, P denotes palindromic repeats, R denotes reverse repeats, and C denotes complementary repeats.

Among the four types of base repeats mentioned above, single base repeats occur the most frequently, with 229 occurrences. Secondly, there are 63 instances of three base repeats; the other base repeats are 17 double base repeats and 6 four base repeats. On the SSR repeat unit type, the number of repetitions of A and T is significantly higher than other types (Figure 3A)

Further analysis shows that among all 315 SSRs, the top three types are T (eight), A (eight), and T (nine), accounting for 15.24% (48), 13.65% (43), and 13.65% (43), respectively (Figure 3A).

Scattered repetitive sequences exist in a dispersed form in the genome. The chloroplast genome of leaf mustard contains 14 forward (F), 18 palindromic (P), three reverse (R), and two complementary (C) sequences, totaling 37 scattered repeat sequences(Table 4). Among them, the length distribution of most scattered repetitive sequences is in the range of 30–58 bp, with the largest number of lengths being 30 bp with ten, followed by 32 bp with five; in addition, there is one scattered repeat sequence with a length of 26,211 bp (Figure 3B).

### 2.5. Nucleic Acid Diversity and Boundary Analysis

**Figure 4:**
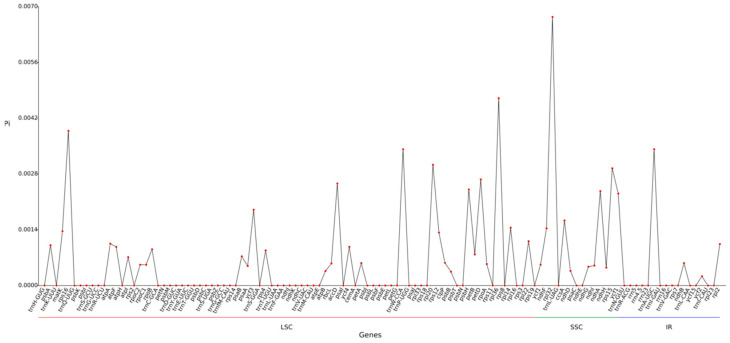
Line chart of gene Pi value.

Pi (nucleic acid diversity) can reveal the magnitude of variation in nucleic acid sequences of different species, and regions with high variability can provide potential molecular markers for population genetics. The analysis of nucleotide diversity in the chloroplast genome of mustard leaves showed that the average nucleic acid diversity (Pi) of all 114 gene regions detected was 0.00059 (Figure 4). From the perspective of regional distribution, the average nucleotide diversity in the SSC region is the highest (0.001409286), followed by the LSC region (0.000509398), and the nucleotide diversity in the IR region is the lowest (0.000308824). The overall Pi value in the IR region is significantly low, reflecting the sequence conservation of this region. The genes contained in IR (such as ribosomal RNA genes) typically have basic and critical functions, and are subject to purification selection constraints, making mutations easily eliminated.

There is no high variability region (Pi ≥ 0.02). The Pi values in the LSC and SSC regions are relatively high, and there are multiple fluctuating peaks, indicating that these two regions are the main enrichment areas of genetic variation. This difference may be related to the diversity of gene functions and heterogeneity of selection pressure within the region.

**Figure 5:**
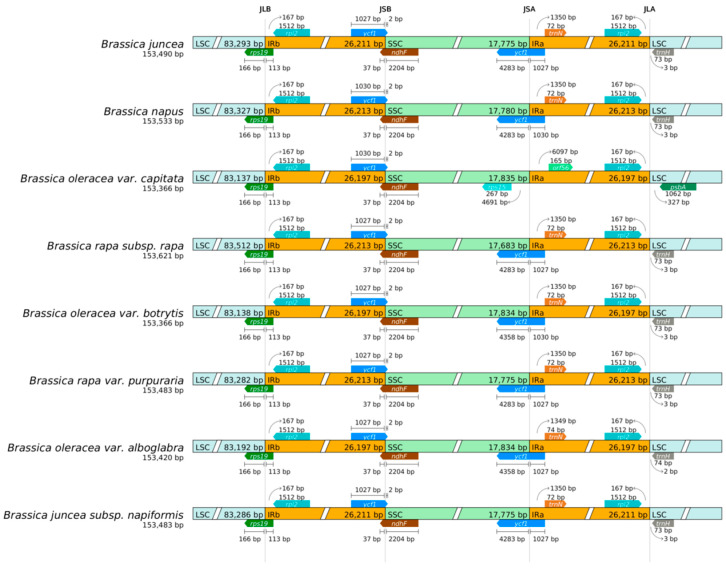
IR/SC boundary analysis.

In the evolutionary process of plant chloroplast genomes, the expansion and contraction of IR boundaries are key factors leading to differences in their size. The analysis of chloroplast genome boundaries of eight cruciferous plants, including mustard greens, showed that there were four boundaries in the chloroplast genomes of these plants, namely JLB (LSC/IRb), JSB (IRb/SSC), JSA (SSC/IRa), and JLA (IRa/LSC). The main genes located near the IR boundary include *rps19*, *rpl2*, *ycf1*, *ndhF*, *trnN*, and *trnH*. Among these eight cruciferous plants, the JLB boundary is located within the coding region of the *rps19* gene, and there is only a 1–2 bp positional difference in the chloroplast genomes of different plants. The JSB boundaries are located within the coding regions of *ycf1* and *ndhF* genes, with an overlap of 36–37 bp between the two. The majority of the *ycf1* gene is located in IRb, with only 2–3 bp in the SSC region; The JSA boundaries are all located within the coding region of the *ycf1* gene, with 1027–1030 bp located in the IRa and 4271–4358 bp located in the SSC; *trnH-GUG* are both located in the LSC, 2–30 bp away from the JLA boundary (Figure 4). The above results show that the chloroplast genomes of eight cruciferous plants, including mustard greens, are highly conserved, and the overall changes in IR boundaries are relatively small, involving only a few genes (Figure 5).

### 2.6. KaKs Analysis

After advanced analysis, using leaf mustard as a comparison, the average Ka/Ks ratio of each gene in the other seven tested species to leaf mustard was 0.16635611.

We analyzed individual genes. Most genes with Ka/Ks < 1 are selected for purification and functionally conserved. Some of these genes are highly conserved when compared to leaf mustard with Ka/Ks = 0. Among the highly variable genes, the *nadhF* gene Ka/Ks of cabbage (KR233156), cauliflower (MT499336), and mustard (OR063916) are 1.28512 (maximum value), while the *petD* gene Ka/Ks of cabbage (KR233156) are 1.16721, all greater than 1, indicating a positive selection effect.

### 2.7. System Evolution Analysis

**Figure 6:**
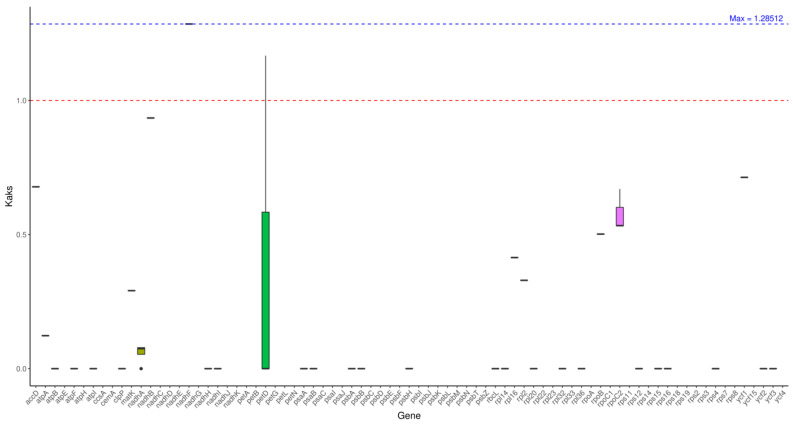
Ka/Ks analysis. Note: The horizontal axis represents gene names, while the vertical axis denotes Ka/Ks ratios. In the box plot, the upper and lower endpoints of the vertical lines above and below the rectangle indicate the upper and lower bounds of the data, respectively. The thick line within the rectangle represents the median, while the upper and lower edges of the rectangle denote the upper and lower quartiles. Data points extending beyond the upper and lower bounds of the rectangle are considered outliers.

In order to gain a more comprehensive understanding of the evolutionary relationships among cruciferous plants, the chloroplast genome data of 15 cruciferous plants were downloaded from the NCBI database. On this basis, a phylogenetic tree was constructed using *Populus adenopoda* Maxim, *Salix babylonica*, and *Gynandropsis gynanda* as outgroups in the family Salicaceae. The results showed that the closest relative to leaf mustard (*Brassica juncea* L.) was *Brassica juncea* subsp. *napiformis*, followed by *Brassica juncea rapa* var. *purpurpuraria*, *Brassica juncea rapa* subsp. *rapa* and other Brassica species. The next closest relative to mustard greens is *Brassica napus*. Finally, there are *Brassica oleracea* var. *capitata*, *Brassica oleracea* var. *botrytis*, and *Brassica oleracea* var. *alboglabra*, all of which belong to the same genus as leaf mustard. Next, radish is closely related to leaf mustard, indicating that in cruciferous plants, radish is an extragenus crop that is evolutionarily similar to other Brassica crops such as leaf mustard. However, the relationship between *Matthiola incana* and *Matthiola longipatala* of the violet genus and leaf mustard is relatively distant, while the *Arabidopsis thaliana*, *Arabidopsis halleri*, *Arabidopsis thaliana*, and the mustard (*Capsella bursa patoris*) and oriental mustard (*Capsella orientalis*) of the Arabidopsis genus have a further relationship with leaf mustard. *Salix babylonica* and *Populus adenopoda*, as outgroups, have the farthest phylogenetic relationship with leaf mustard (Figure 6).

**Figure 7:**
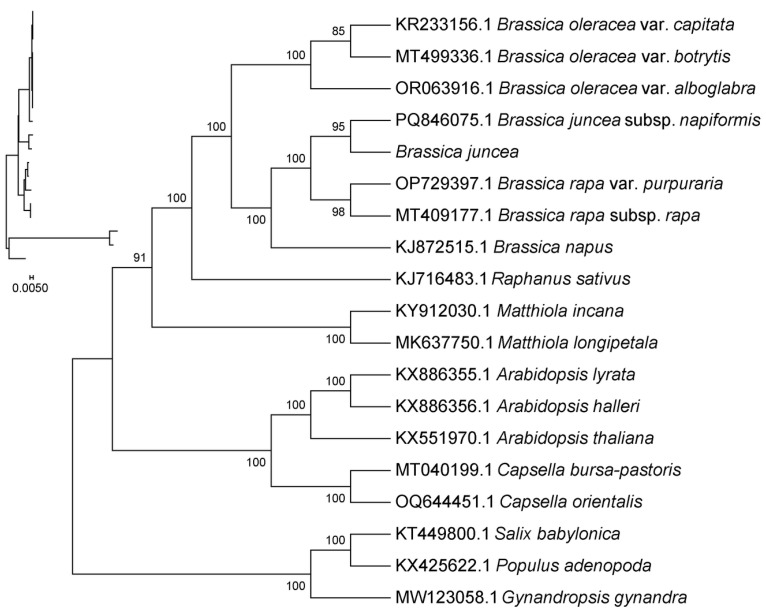
Phylogenetic tree constructed based on chloroplast genome sequences. Note: (1) Sequence names correspond to species Latin names. (2) Branch length: Also known as genetic variation or evolutionary distance. Represents the degree of change in evolutionary branches; shorter lengths indicate smaller differences and closer evolutionary distances. (3) Distance scale: The unit length for measuring differences between organisms or sequences, equivalent to the scale of an evolutionary tree. (4) Self-expansion value: Used to display the reliability of evolutionary tree branches. Typically represented by a number between 0 and 100.

In addition, the results showed that the mustard greens used for leaves were selected from the same cruciferous species as the white cauliflower (*Gynandropsis yunandra*), but the genetic relationship between the two was relatively distant, indicating a complex composition of cruciferous species(Figure 7).

## 3. Discussion

The chloroplast genome of leaf mustard assembled in this study is 153,490 bp in length and exhibits a typical tetrad structure, which is consistent with the chloroplast genome characteristics of most terrestrial plants. Its GC content (36.36%) is consistent with the GC content of mustard greens, nori stems, European rapeseed, cabbage, cauliflower, and mustard greens, all of which belong to the Brassica genus. From this, it can be seen that the chloroplast genomes of various species in the mustard and Brassica genera are relatively conserved, which is consistent with the research results of ZHAO et al. [14].

The annotation of the chloroplast genome of mustard leaves showed 132 coding genes, and no pseudogenes were found. Among them, 44 genes were related to photosynthesis. Compared with published species of Brassica, their gene types and quantities showed significant differences, indicating that the core functional genes of the chloroplast genome were highly conserved during evolution [15]. In the process of plant evolution, the use of different codons usually exhibits certain preferences. In the chloroplast genome of mustard greens, the most frequently used amino acid is leucine (Leu), and the least frequently used amino acid is cysteine (Cys), which is consistent with the research results of plants such as *Ananas comosus* var. *comosus* [16] and *Magnolia zenii* [17]. Relative synonymous codon usage frequency (RSCU) is defined by comparing the actual frequency of occurrence of a specific codon with its theoretical expected frequency, and is an effective tool for evaluating codon bias. An RSCU greater than 1 indicates a clear preference for the use of that codon [18]. In the chloroplast genome of leaf mustard, the vast majority (93.55%) of codons with an RSCU greater than 1 end in A or U, while the vast majority (93.55%) of codons with an RSCU less than 1 end in A or U. Similar phenomena are commonly found in the chloroplast genome of angiosperms—for example, among the 67 coding sequences in the chloroplast genome of cauliflower, there are 32 codons with RSCU values greater than 1.00, which are high-frequency codons. Among them, 13 codons end in A, 16 codons end in U, and the remaining three codons end in G. Synonymous codons ending in A or U account for 90.6%; among the 34 codons with RSCU values less than 1.00, 13 end in G, 17 end in C, and 4 end in A. Codons ending in G or C account for 88.2%. There is a codon RSCU value equal to 1—indicating a high degree of conservation in the frequency of codon usage in the chloroplast genome [19,20]. SSRs are widely used for constructing genetic linkage maps, population genetic analysis, and more. ZHAO et al. [14] identified a total of 290 SSRs in the chloroplast genome of *Brassica oleracea* var. *gongylodes*, while WU et al. [21] discovered 288 SSRs in winter rapeseed. In this study, a total of 315 SSRs were found in the chloroplast genome of leaf mustard, providing potential candidate molecular markers for studying the genetic diversity of Brassica crops.

Nucleotide diversity is an important indicator used to measure the degree of genetic variation within a population. The higher its value, the richer the genetic diversity within the population, which can provide potential molecular markers for population genetics [22]. This study compared and analyzed eight species of cruciferous plants, including mustard and nori, and found that the average nucleotide diversity of the chloroplast genomes of the eight species was 0.00059. The average nucleotide diversity in different regions, from highest to lowest, was the SSC (0.001409286), LSC (0.00509398), and IR (0.000308824), indicating that the IR is more conserved compared to the other two regions; The top four sites with the highest nucleotide diversity are *rpl32* in the SSC region, *rpl36* and *rps16* in the LSC region, and *trnA-UGC* in the IR region. These highly variable sites can be used as molecular markers for species identification in the Brassicaceae family. In the process of plant genome evolution, the expansion or contraction of the IR region is the main driving force of chloroplast genome structural variation, which can provide a molecular basis for species identification and phylogenetic research [23]. Analysis of the IR boundaries of eight cruciferous species, including mustard greens, revealed that the differences in IR boundaries were mainly related to the positions of *rps19*, *ycf1*, *ndhF*, and *trnH*, but the overall changes were relatively small, indicating that the chloroplast genomes of cruciferous plants such as mustard greens are relatively conserved.

As the second largest genome in plants, the chloroplast genome exhibits significant differences in the evolutionary rates of its coding and non-coding regions, making it suitable for systematic research at different levels. It has been widely used in plant phylogenetic reconstruction and population analysis [24]. Based on the chloroplast genome, the phylogenetic analysis of leaf mustard using the maximum likelihood method provides a basis for the evolutionary position of leaf mustard in the Brassicaceae family. The results of this study showed that mustard greens have the closest genetic relationship, belonging to the hybrid of black mustard and Brassica napus, followed by Brassica vegetables such as nori and turnip, European rapeseed and other hybrid vegetables of Brassica napus, and finally cabbage, cauliflower, mustard greens and other Brassica vegetables.

## 4. Materials and Methods

### 4.1. Test Materials and Sequencing

**Figure 8:**
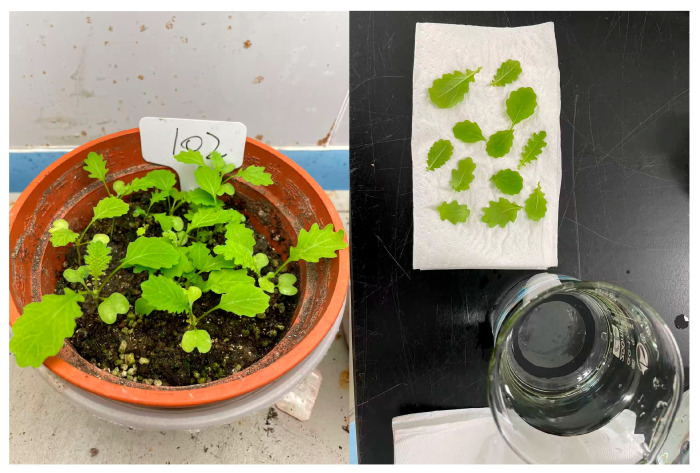
Two photographs of the plant material (“Chicken Crown Snow Cabbage” leaf mustard).

The material used in this experiment is ‘Chicken Crown Snow Cabbage’ leaf mustard, which was planted at the Zhejiang Institute of Landscape Plants and Flowers (Xiaoshan Cotton and Hemp Research Institute, Hangzhou, Zhejiang Province, China) (30°07′ N, 120°23′ E). The tender leaves of healthy plants were taken, cleaned and dried without residue. After sampling, they were frozen in liquid nitrogen and placed in a pre-cooled EP tube for 10 min. After removal, they were stored in a −80 °C freezer (Figure 8). Total genomic DNA was extracted from the leaf samples using a universal plant DNA extraction kit (Genepioneer, D312). Paired-end (PE) sequencing was performed on the Illumina NovaSeq 6000 platform.

### 4.2. Chloroplast Genome Assembly and Functional Annotation

Raw sequencing data were filtered using fastp v0.23.4 [25] to remove sequencing adapters and primer sequences from the reads. Reads with an average quality score below Q5 or containing more than 5 ambiguous bases (N) were discarded, resulting in clean data. The chloroplast genome was assembled using GetOrganelle v1.7.7.1. To improve annotation accuracy, two complementary methods were employed for functional annotation. First, prodigal v2.6.3 [26] was used to predict protein-coding sequences (CDSs), hmmer v3.1b2 [27] was used to identify rRNA genes, and ARAGON v1.2.38 [28] was used to predict tRNA genes. Second, the assembled sequence was compared with the gene sequences of closely related species publicly available on NCBI using BLAST v2.6 [29] to generate a second set of annotations. The two annotation sets were then manually inspected to resolve discrepancies, remove erroneous and redundant annotations, and confirm the boundaries of multi-exon genes, yielding the final, curated annotation. A graphical map of the chloroplast genome was generated using OGDRAW [30].

### 4.3. Analysis of Scattered Repetitive Sequence and Simple Repetitive Sequence

Dispersed repeat sequences were identified using the vmatch v2.3.0 software [31] in conjunction with custom Perl scripts. Its parameter settings were: minimum length = 30 bp, and Hamming distance = 3, and there were four identification forms: forward, palindromic, reverse, and complementary. Chloroplast simple sequence repeats (cpSSRs) were detected using the MISA v1.0 software [32] with parameters 1–8 (single base repeat 8 times or more), 2-5, 3-3, 4-3, 5-3, and 6-3.

### 4.4. Chloroplast Genomic Nucleic Acid Diversity and Boundary Analysis

The complete chloroplast genomes of eight Brassicaceae species were downloaded from the NCBI database, including *Brassica juncea* L., *Brassica juncea* subsp. *Napiformis* (PQ846075.1), *Brassica juncea rapa* var. *purpuraria* (OP729397.1), *Brassica juncea rapa* subsp. *Rapa;* MT409177.1), *Brassica napus* (KJ872515.1), *Brassica oleracea* var. *capitata*(KR233156.1), *Brassica oleracea* var. *botrytis* (MT499336.1) and *Brassica oleracea* var. *alboglabra* (OR063916.1). The mafft software (v7.427, auto mode) was used to globally align homologous gene sequences of different species, and calculate the pi value of each gene using dnasp5 [33]. Using the cloud platform tool CPJSdraw from Jisihuiyuan (http://cloud.genepioneer.com:9929/#/tool/alltool/detail/296, accessed on 15 October 2025), we visualized the boundary information and per- formed genome alignment using the default parameters of Mauve (v2.3.1) [34] software.

### 4.5. System Evolution Analysis

For phylogenetic analysis, the complete chloroplast genome sequences of 16 species from the order Brassicales were downloaded from the NCBI database. Three species, Salix babylonica, Populus adenopoda, and Gynandropsis gynandra, were selected as outgroups. Phylogenetic tree analysis based on shared CDS sequences: First, multiple sequence alignment was performed using MAFFT v7.427 (auto mode), and unreliable alignment regions were removed using trimAl (v1.4. rev15) [35] and species CDS sequences were concatenated; jModelTest v2.1.10 was reused to screen the optimal nucleotide substitution model under Bayesian information criteria; and finally, RAxML v8.2.10 [36] was used to construct a maximum likelihood evolutionary tree using the GTRGAMMA model and 1000 rapid bootstrap analyses.

## 5. Conclusions

This study identified the basic characteristics of the chloroplast genome of leaf mustard, which is 153,490 bp in length with a typical tetrad structure. It has a GC content of 36.36% and encodes 132 genes, including 87 protein coding genes, 37 tRNA genes, and eight rRNA genes. The gene functions mainly include photosynthesis and self-replication. Its genome codons mostly end in A/U. The repetitive sequences are mainly single nucleotide repeats, with a total of 37 scattered repetitive sequences and 315 simple repetitive sequences. Nucleic acid diversity analysis revealed that the SSC region had the highest variation, the IR region was the most conservative, and there were no high variation regions. IR boundary analysis shows that the boundary structure of Brassica plants is generally conservative, with only minor differences. The phylogenetic tree indicates that the leaf mustard has the closest evolutionary relationship with plants of the same genus, and is relatively close to the radish and violet genera, providing molecular evidence for the classification and evolutionary research of the Brassicaceae family.

The results of this study supplement the basic data of the chloroplast genome of leaf mustard, providing an effective tool for the identification and genetic improvement of leaf mustard germplasm resources, and offering a new perspective for the study of evolutionary relationships in cruciferous plants.

## Figures and Tables

**Table 1:** Base composition characteristics of different sequence regions (LSC, SSC, IRa, IRb).

Sequence Type Characteristics	Base Type	Number	%
Large-Scale Copy Region (LSC) Feature	A	26,695	32.05%
	C	14,613	17.54%
	G	13,806	16.58%
	T	28,179	33.83%
	GC	28,419	34.12%
	All	83,293	100.00%
Small-Scale Copy Region (SSC) Feature	A	6309	35.49%
	C	2695	15.16%
	G	2496	14.04%
	T	6275	35.30%
	GC	5191	29.20%
	All	17,775	100.00%
Inverse Repeat Sequence a (IRa) Feature	A	7575	28.90%
	C	5775	22.03%
	G	5323	20.31%
	T	7538	28.76%
	GC	11,098	42.34%
	All	26,211	100.00%
Inverse Repeat Sequence b (IRb) Feature	A	7538	28.76%
	C	5323	20.31%
	G	5775	22.03%
	T	7575	28.90%
	GC	11,098	42.34%
	All	26,211	100.00%
Total Amount	A	48,117	31.35%
	C	28,406	18.51%
	G	27,400	17.85%
	T	49,567	32.29%
	GC	55,806	36.36%
	All	153,490	100.00%

**Table 2:** Gene annotation of the chloroplast genome of *Brassica juncea* L.

Category	Gene Group	Gene Name
Photosynthesis	Subunits of photosystem I	*psaA*, *psaB*, *psaC*, *psaI*, *psaJ*
	Subunits of photosystem II	*psbA*, *psbB*, *psbC*, *psbD*, *psbE*, *psbF*, *psbH*, *psbI*, *psbJ*, *psbK*, *psbL*, *psbM*, *psbN*, *psbT*, *psbZ*
	Subunits of NADH dehydrogenase	ndhA *, ndhB * (2), *ndhC*, *ndhD*, *ndhE*, *ndhF*, *ndhG*, *ndhH*, *ndhI*, *ndhJ*, *ndhK*
	Subunits of cytochrome b/f complex	*petA*, petB *, petD *, *petG*, *petL*, *petN*
	Subunits of ATP synthase	*atpA*, *atpB*, *atpE*, atpF *, *atpH*, *atpI*
	Large subunit of rubisco	*rbcL*
	Subunits photochlorophyllide reductase	*-*
Self-replication	Proteins of large ribosomal subunit	*rpl14*, rpl16 *, rpl2 * (2), *rpl20*, *rpl22*, *rpl23(2)*, *rpl32*, *rpl33*, *rpl36*
	Proteins of small ribosomal subunit	*rps11*, rps12 ** (2), *rps14*, *rps15*, rps16 *, *rps18*, *rps19*, *rps2*, *rps3*, *rps4*, *rps7(2)*, *rps8*
	Subunits of RNA polymerase	*rpoA*, *rpoB*, rpoC1 *, *rpoC2*
	Ribosomal RNAs	*rrn16(2)*, *rrn23(2)*, *rrn4.5(2)*, *rrn5(2)*
	Transfer RNAs	trnA-UGC * (2), *trnC-GCA*, *trnD-GUC*, *trnE-UUC*, *trnF-GAA*, *trnG-GCC*, trnG-UCC *, *trnH-GUG*, *trnI-CAU(2)*, trnI-GAU * (2), trnK-UUU *, *trnL-CAA(2)*, trnL-UAA *, *trnL-UAG*, *trnM-CAU*, *trnN-GUU(2)*, *trnP-UGG*, *trnQ-UUG*, *trnR-ACG(2)*, *trnR-UCU*, *trnS-GCU*, *trnS-GGA*, *trnS-UGA*, *trnT-GGU*, *trnT-UGU*, *trnV-GAC(2)*, trnV-UAC *, *trnW-CCA*, *trnY-GUA*, *trnfM-CAU*
Other genes	Maturase	*matK*
	Protease	clpP **
	Envelope membrane protein	*cemA*
	Acetyl-CoA carboxylase	*accD*
	c-type cytochrome synthesis gene	*ccsA*
	Translation initiation factor	*-*
	other	*-*
Genes of unknown function	Conserved hypothetical chloroplast ORF.	*ycf1(2)*, *ycf15(2)*, *ycf2(2)*, ycf3 **, *ycf4*

Note: Gene *: contains one intron; Gene **: contains two introns; Gene: pseudogene; Gene (2): gene with copy number greater than 1, with copy number indicated in parentheses.

**Table 3:** Relative synonymous codon usage analysis of *Brassica juncea* L.

Symbol	Codon	No.	RSCU	Symbol	Codon	No.	RSCU	Symbol	Codon	No.	RSCU
Ter *	UAA	48	1.8228	Ile	AUA	624	0.9474	Pro	CCC	161	0.7024
Ter *	UAG	19	0.7215	Ile	AUC	350	0.5313	Pro	CCG	124	0.5408
Ter *	UGA	12	0.4557	Ile	AUU	1002	1.5213	Pro	CCU	366	1.5964
Ala	GCA	348	1.1208	Lys	AAA	995	1.5644	Gln	CAA	641	1.571
Ala	GCC	177	0.57	Lys	AAG	277	0.4356	Gln	CAG	175	0.429
Ala	GCG	131	0.422	Leu	CUA	323	0.8022	Arg	AGA	384	1.755
Ala	GCU	586	1.8872	Leu	CUC	149	0.3702	Arg	AGG	126	0.576
Cys	UGC	62	0.461	Leu	CUG	138	0.3426	Arg	CGA	296	1.3524
Cys	UGU	207	1.539	Leu	CUU	497	1.2342	Arg	CGC	96	0.4386
Asp	GAC	169	0.3898	Leu	UUA	871	2.163	Arg	CGG	102	0.4662
Asp	GAU	698	1.6102	Leu	UUG	438	1.0878	Arg	CGU	309	1.4118
Glu	GAA	928	1.5492	Met	AUA	0	0	Ser	AGC	100	0.354
Glu	GAG	270	0.4508	Met	AUC	0	0	Ser	AGU	355	1.2576
Phe	UUC	401	0.5876	Met	AUG	512	6.9867	Ser	UCA	339	1.2006
Phe	UUU	964	1.4124	Met	AUU	0	0	Ser	UCC	235	0.8322
Gly	GGA	624	1.6104	Met	CUG	0	0	Ser	UCG	156	0.5526
Gly	GGC	153	0.3948	Met	GUG	1	0.0133	Ser	UCU	509	1.803
Gly	GGG	254	0.6556	Met	UUG	0	0	Thr	ACA	366	1.2344
Gly	GGU	519	1.3392	Asn	AAC	244	0.444	Thr	ACC	211	0.7116
His	CAC	125	0.4922	Asn	AAU	855	1.556	Thr	ACG	121	0.408
His	CAU	383	1.5078	Pro	CCA	266	1.1604	Thr	ACU	488	1.646
								Val	GUA	455	1.456
								Val	GUC	152	0.4864
								Val	GUG	168	0.5376
								Val	GUU	475	1.52
								Trp	UGG	396	1
								Tyr	UAC	155	0.3694
								Tyr	UAU	684	1.6306

Note: Symbols: Three-letter amino acid abbreviation, ‘*’ denotes stop codon; Codon: codon; No.: number of codons; RSCU: codon preference.

**Table 4:** Analysis of scattered sequence repeats in the chloroplast genome of *Brassica juncea* L.

Length	F	P	R	C	Total
30	3	7	0	1	11
31	1	0	2	0	3
32	3	2	0	0	5
33	0	1	0	0	1
34	2	2	0	0	4
35	0	0	1	0	1
36	0	1	0	0	1
37	1	1	0	1	3
40	0	1	0	0	1
42	1	0	0	0	1
43	1	0	0	0	1
44	0	1	0	0	1
45	0	1	0	0	1
46	1	0	0	0	1
58	1	0	0	0	1
26,211	0	1	0	0	1
Total	14	18	3	2	37

## Data Availability

All original data (including sequencing reads and annotated genomes) supporting the reported results have been submitted to NCBI GenBank (PubMed). The corresponding GenBank accession numbers will be provided once they are assigned, and the data can be accessed via the NCBI platform.
